# Sex Differences in Age-Related Functional Mobility Decline: A Moderated Mediation Analysis

**DOI:** 10.3390/sports14070270

**Published:** 2026-06-30

**Authors:** Filipe Rodrigues, Vasco Bastos, Diogo Santos Teixeira, Diogo Monteiro

**Affiliations:** 1 ESECS–Polytechnic University of Leiria, Campus 1, Rua Dr. João Soares, Apartado 4045, 2411-901 Leiria, Portugal; diogo.monteiro@ipleiria.pt; 2 Research Center in Sports Sciences, Health Sciences and Human Development (CIDESD), Quinta de Prados, 5001-801 Vila Real, Portugal; 3 Faculty of Physical Education and Sport–Lusófona University, Campo Grande 376, 1749-024 Lisboa, Portugal; vasco.bastos@ulusofona.pt (V.B.); diogo.teixeira@ulusofona.pt (D.S.T.); 4 Research Center in Sports, Physical Education, Exercise and Health (CIDEFES), Campo Grande 376, 1749-024 Lisboa, Portugal

**Keywords:** sarcopenia, functional mobility, aging, sex characteristics, muscle strength

## Abstract

While functional mobility is known to deteriorate with age, the specific mechanisms driving this decline, along with potential differences between men and women, are not fully established. Therefore, the present study applied a moderated mediation framework to investigate whether lower limb strength and body mass index (BMI) mediate the relationship between aging and functional mobility, and to explore if sex acts as a moderator in these relationships. A cross-sectional design was used to evaluate 408 independent older adults (mean age: 71.9 ± 3.9 years; 273 females and 135 males). Assessments included the Timed Up and Go (TUG) for functional mobility, the 30 s Chair Stand for leg strength, alongside BMI calculations. Direct and indirect pathways were subsequently evaluated using a moderated mediation analysis. Lower limb strength and BMI significantly predicted functional mobility in both sexes (*p* < 0.05), with no significant moderation in the indirect pathways. Regarding conditional effects, advancing age predicted poorer functional mobility in females (*p* = 0.008) but not in males (*p* = 0.141), independent of the mediators. Strength and BMI appear to be correlates of functional mobility. However, since the age-by-sex interaction term was not statistically significant, the potential sex-specific direct effect of age must be interpreted with caution. While these findings hint at possible subtle differences in aging trajectories, future research is needed to confirm whether interventions for older women require addressing factors beyond strength and body composition.

## 1. Introduction

Accelerated demographic aging poses significant challenges to healthcare systems, with impaired balance and subsequent falls representing one of the leading causes of morbidity and mortality in older populations [1]. This epidemiological trend is characterized by alarming prevalence rates in community-dwelling older adults [2], often exacerbated by concerned levels of sedentary behavior and muscle frailty [3]. The precise characterization of this phenomenon is not merely a clinical necessity but an economic imperative, given that prevention-focused interventions have demonstrated a favorable cost-utility ratio in reducing hospital expenditures [4]. Furthermore, the prevalence of sarcopenia among older adults suggests that the loss of muscle function is a central determinant in the etiology of these events [5].

Regarding fall risk assessment, the Timed Up and Go (TUG) test remains the gold standard for evaluating basic functional mobility [6], presenting robust predictive capacity for future fall events [7]. Recent investigations from 2025 continue to validate the clinical utility of the TUG and refine its cut-off points for specific subpopulations [8], underscoring its relevance in monitoring functional mobility [9]. However, the deterioration of TUG performance is not uniform. Literature indicates that the age-related decline in functional mobility is mediated by complex physiological processes and displays distinct trajectories between males and females [10,11], suggesting a sexual dimorphism in aging mechanisms.

The revised European consensus on sarcopenia established muscle strength as the primary determinant of sarcopenia [12]. Emerging evidence, including recent meta-analyses from 2025, corroborates that reduced strength is an independent and potent predictor of falls [13]. While strength markers ranging from handgrip strength [14,15] to toe grip strength [16] have been associated with balance, assessments of lower limb strength offer greater functional relevance for locomotion and stability. In particular, the 30 s chair stand test evaluates the muscles responsible for resisting gravity, which are crucial for preventing falls. This assessment features established normative data [17,18] and has consistently demonstrated strong reliability in identifying functional deterioration among older populations, whether they live independently or in institutions [19,20,21].

Furthermore, body mass index (BMI) as a measure of body composition serves a vital, albeit complex, function. While carrying excess weight correlates with an elevated fall risk [22] and diminished functional performance [23], extensive recent research continues to question the ideal BMI thresholds needed to maintain physical autonomy. This underscores the intricate link between functional decline and body composition [24,25]. The coexistence of low muscle mass and high adipose tissue, phenotypically described as sarcopenic obesity, represents a scenario of aggravated risk for mortality and physical disability [26,27]. Factors such as sedentary behavior exacerbate this condition [28], necessitating the consideration of functional benchmark [29], or the interaction between mobility and mortality risk in predicting functional capacity [30].

Despite the known efficacy of multicomponent physical exercise in reversing frailty [31] and mitigating fall risk across various frailty stages [32], gaps persist in understanding the exact mechanisms precipitating functional decline. Previous studies predominantly focused on direct associations or linear predictions of physical performance [33,34,35]. However, the interplay between these factors is likely complex and sex-specific. Theoretically, sex could moderate the effect of age on body composition and strength (e.g., due to post-menopausal hormonal shifts), the impact of these physical mediators on mobility (e.g., due to sexual dimorphism in fat distribution and biomechanics), and the direct trajectory of age-related balance decline (e.g., influenced by distinct neurosensory or behavioral adaptations, such as fear of falling). Consequently, current research, driven by new data on physical activity levels [36] and updated strength parameters [37], may demand more sophisticated statistical models. Our recent line of research has sought to map these variables in the older population [38,39,40]. The present study aims to expand this knowledge by testing a moderated mediation model to determine whether the effect of age on functional mobility is mediated by lower limb strength and BMI, and whether these mechanisms operate with distinct magnitudes depending on sex.

## 2. Materials and Methods

### 2.1. Participants

The statistical power of the final sample size (N = 408) was verified using a post-hoc power analysis for moderated mediation via the WebPower (ISDSA Press, Granger, IN, USA) platform [41]. The simulation parameters were configured for a parallel moderated mediation structure based on standard reference parameters for this framework, with a significance level (α) of 0.05. The analysis indicated that the sample of 408 participants provided an adequate and robust statistical power to evaluate the conditional indirect effects and moderation pathways. This mathematical verification supports the methodological adequacy of our sample size to analyze the hypothesized relationships within the model.

The study sample comprised volunteers aged 65 years and older, recruited from the community setting. Eligibility was contingent upon the provision of written informed consent, medical clearance for physical assessment, and the ability to ambulate independently without assistive devices. Participants were required to demonstrate adequate cognitive and sensory function to comply with the testing protocols. Exclusion criteria encompassed diagnosed neurological or cognitive disorders affecting motor control.

### 2.2. Procedures

All ethical guidelines established by the Declaration of Helsinki were rigorously followed, and the research protocol was cleared by the Ethics Committee of the Polytechnic of Leiria (reference: CE/IPLEIRIA/63/2024; approved 24 May 2024). We utilized a convenience sample, selecting individuals from community-based exercise programs. The recruitment pathway was hierarchical: initial permission was granted by facility directors, followed by staff assistance in pinpointing suitable candidates. Before any procedures, participants were informed about the confidentiality and voluntary nature of the research, subsequently providing their written informed consent.

The assessment phase began with the collection of demographic data, where participant sex (male or female) was self-reported, followed by anthropometrics. Standing height was obtained to the closest 0.1 cm via a portable stadiometer (Seca 213, Hamburg, Germany), ensuring the head was aligned in the Frankfurt plane. A digital scale (Seca 813, Hamburg, Germany) captured body mass to the nearest 0.1 kg, and these metrics allowed for BMI calculation. Physical function was then evaluated according to the Senior Fitness Test guidelines [17]. To measure lower-body strength, we utilized the 30-Second Chair Stand Test. Subjects sat in an armless chair with feet planted and arms folded across the chest, completing as many full stands and returns to the seated position as possible within a 30 s window. The final count of valid cycles was registered. Finally, functional mobility and agility were evaluated using the TUG test. Standardized verbal instructions were provided, directing participants to stand up, walk as quickly and safely as possible around the cone, and return to a fully seated position. After viewing a demonstration and completing a familiarization trial, subjects rose from a chair, navigated 2.44 m to round a cone, and returned to sit down. In instances of protocol violations, such as failing to complete the exact trajectory, running, or inappropriate use of physical support, the trial was immediately halted, and the participant was allowed a brief rest before repeating the attempt. Participants performed two valid timed trials at a safe but rapid pace, monitored with a stopwatch (0.01 s precision). The fastest of the two attempts was retained for the final analysis.

### 2.3. Statistical Analysis

Data were analyzed utilizing IBM SPSS Statistics v31.0 (IBM Corp., Armonk, NY, USA), with the threshold for statistical significance established at *p* < 0.05. To outline the participant profile, standard descriptive metrics were computed, namely frequencies, means, and standard deviations. The assumption of normality was verified by combining the Kolmogorov–Smirnov test with visual histogram evaluations. Finally, the Hayes PROCESS macro (Version 5.0) [42] for SPSS was employed to execute the moderated mediation analysis and evaluate the conceptual pathways.

To test the proposed conceptual model, a moderated mediation analysis was conducted using the PROCESS macro for SPSS (Version 5.0), developed by Hayes [42]. Specifically, Model 59 was employed to examine whether the indirect and direct effects of Age (X) on the TUG performance (Y) through Chair Stand performance (M1) and BMI (M2) were moderated by Sex (W). In this model, age was entered as the independent variable, TUG as the dependent variable, and both Chair Stand repetitions and BMI as parallel mediators. Sex was included as the moderator, hypothesized to influence all three pathways: (a) the effect of age on the mediators (first stage of mediation), (b) the effect of the mediators on TUG (second stage of mediation), and (c) the direct effect of age on TUG (direct path). All continuous variables that defined the products were mean centered to avoid multicollinearity [43]. Bootstrapping techniques utilizing 5000 resamples were employed to assess the significance of conditional indirect pathways by calculating 95% bias-corrected confidence intervals (CIs). A specific effect was deemed statistically meaningful whenever zero fell outside the resulting confidence interval. Additionally, we calculated the index of moderated mediation to ascertain whether the strength of these mediating pathways varied significantly between male and female participants.

## 3. Results

Table 1 details the sample’s baseline profiles and bivariate relationships, separated by sex. Correlational data highlighted similar trends for both men and women concerning body composition and functional capacity. Notably, chair stand scores demonstrated a significant inverse relationship with both TUG times (*p* < 0.01) and BMI (*p* < 0.01) across sexes, suggesting that robust leg strength aligns with enhanced agility and lower relative body mass. Furthermore, elevated BMI values were strongly linked to prolonged TUG completion times (*p* < 0.01). Interestingly, chronological age showed no significant associations with either BMI or functional outcomes within this specific cohort.

**Figure 1:**
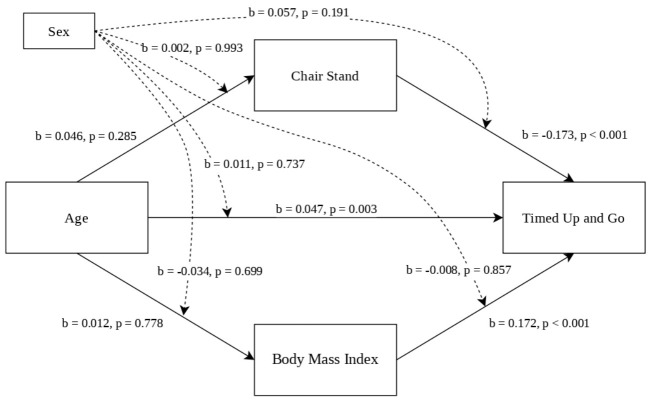
Conceptual diagram of the moderated mediation analysis. Values presented on the pathways represent unstandardized regression coefficients (*b*) and significance levels (*p*) for males and females separately. Curved arrows represent the moderating effect of Sex on each path, with the corresponding *p*-values for the interaction terms.

The effects of age and sex on the mediators were tested, as depicted in Figure 1. The comprehensive regression results of the moderated mediation analysis, including model summary statistics, standard errors, and confidence intervals, are detailed in Table 2. The regression model predicting functional mobility was statistically significant (R^2^ = 0.3699, F = 33.55, *p* < 0.001). Both mediators significantly predicted timed up and go performance: chair stand performance negatively predicted timed up and go performance (*b* = −0.1732, *p* < 0.001), and body mass index positively predicted TUG time (*b* = 0.1722, *p* < 0.001).

Regarding the moderation hypotheses, the interaction terms representing the moderating effect of sex were not statistically significant for any of the pathways: age × sex on TUG (*b* = 0.011, *p* = 0.739), chair stand × sex on TUG (*b* = 0.057, *p* = 0.191), and BMI × sex on TUG (*b* = −0.008, *p* = 0.857). Despite the non-significant interaction term for the direct effect, the analysis of conditional effects revealed a trend worthy of note. The direct effect of age on TUG was statistically significant for females (*b* = 0.050, *p* = 0.008), but not for males (*b* = 0.039, *p* = 0.141). However, given that the numerical difference between these coefficients is very small (0.011) and the interaction term is non-significant, these findings do not support a clear sex-specific dimorphism and require replication in a longitudinal design. This suggests that, within this specific cohort, advancing age presents a detectable statistical association with slower TUG performance in females, independent of BMI or strength levels.

Regarding the mediation pathways, the indirect effect of age on functional mobility through lower limb strength was not statistically significant for either sex (Males: *b* = −0.0087, 95% CI [−0.0364, 0.0209]; Females: *b* = −0.0074, 95% CI [−0.0252, 0.0083]). Similarly, the indirect pathway through BMI showed no significant mediation for males (*b* = 0.0061, 95% CI [−0.0161, 0.0355]) or females (*b* = 0.0001, 95% CI [−0.0176, 0.0177]). These findings reinforce that neither strength nor BMI significantly mediates the relationship between age and functional mobility in this cohort. As all bootstrap confidence intervals included zero, the hypothesized mediation pathways were not supported by the data for either sex.

## 4. Discussion

This research sought to explore the factors behind the deterioration of functional mobility as people age. To achieve this, we evaluated a moderated mediation framework incorporating body mass index (BMI) and leg strength as variables across both sexes. The central finding of this investigation is twofold. First, lower limb strength and BMI act as predictors of functional mobility, operating with similar magnitude in both males and females. Second, while conditional analysis showed that the direct effect of age on functional mobility was significant for females but not for males, the overall age-by-sex interaction was not significant. This indicates that evidence for true sex differences in this pathway may be weak. Furthermore, sex did not significantly moderate the indirect pathways. As age was not significantly associated with chair stand performance or BMI, the hypothesized indirect pathways were not supported by our data. This suggests that rather than acting as mediators of the aging process, muscle capacity and body composition operate as strong, independent predictors of functional mobility in this age cohort, with their importance being conserved across sexes, and any assumptions regarding distinct sex-specific aging trajectories must be made cautiously.

Our results reinforce the revised European consensus on sarcopenia [12], identifying muscle strength as a critical determinant of function. The negative association found between chair stand performance and TUG time confirms that maintaining force-generating capacity is the primary buffer against impaired balance, regardless of sex. In a biomechanical context, the TUG requires the ability to generate explosive power to rise from a seated position, accelerate, and decelerate during the turn. Our data indicates that this requirement is considerably important. A specific threshold of lower limb strength is necessary to execute these motor patterns efficiently, and when this strength is compromised, functional mobility declines linearly in both males and females. Similarly, the detrimental effect of BMI on TUG performance was evident in both groups. This supports the “mechanical constraint” hypothesis, where excess body mass increases the inertial load required to perform dynamic tasks [23]. This creates a demand for greater relative strength, a demand that aging muscles often fail to meet. This clinical implication aligns with the emerging concept of powerpenia, recently highlighted by Freitas et al. [44], which suggests that the age-related loss of muscle power, specifically relative to body mass (power-to-weight ratio), may be a more critical determinant of functional performance than the loss of muscle mass alone. The absence of sex moderation in this pathway suggests that the harmful physics of excess adiposity on functional mobility affects male and female physiology with equal severity.

An exploratory finding from our conditional analysis is that advancing age was directly associated with poorer functional mobility in females (*p* = 0.008) but not in males (*p* = 0.141), even after statistically adjusting for strength and BMI levels. Although the lack of a significant interaction term requires a cautious interpretation, this conditional effect hints that, for females, the decline in functional mobility might involve latent factors beyond simple muscle weakness or weight gain. Although our study did not assess these variables, we hypothesize that several unmeasured physiological and behavioral mechanisms could theoretically explain this residual direct effect. First, as a speculative factor, the post-menopausal decline in estrogen is linked not only to muscle quality but also to a deterioration in vestibular and proprioceptive inputs, which are essential for the turning component of the TUG [10]. The integration of sensory information is paramount for stability during the transition phases of gait, and females in this age bracket may experience a more pronounced degradation of these neurosensory pathways compared to their male counterparts. Furthermore, a metabolic perspective offers another plausible explanation. Since BMI does not distinguish between lean mass and adipose tissue, it may fail to capture sex-specific changes in body composition. It is possible that the post-menopausal period induces a more accelerated trajectory of sarcopenic obesity in women, characterized by intramuscular fat infiltration not detected by BMI, compared to men. This qualitative deterioration of muscle would explain why age predicts poorer balance in women even when quantitative BMI is controlled. Second, although hypothetical in the context of our data, behavioral adaptations to aging must be considered. Older females typically exhibit higher rates of fear of falling compared to males [15]. This psychological constraint often manifests physically as a “cautious gait” strategy, characterized by shorter stride length, wider base of support, and prolonged double-support time, which inevitably inflates TUG scores independent of the individual’s physiological capacity to move faster. In contrast, the male participants in our sample, representing community-dwelling older adults, may possess a physiological or neural reserve that delays the direct impact of chronological age on balance until later life stages, or they may simply exhibit a less conservative motor strategy. However, we emphasize that because these hormonal, metabolic, and psychological factors were not directly measured in our study, these interpretations remain entirely speculative and highlight key targets for future targeted research.

It is also noteworthy that age did not correlate with functional outcomes (as shown in Table 1) and was not a significant predictor of chair stand performance or BMI in our model. While this defies the classic trajectory of age-related functional decline, it may be explained by the specific bio-behavioral characteristics of our sample. By recruiting from community-based physical activity programs and strictly excluding those with recurrent falls or neurological conditions, we likely selected a successful aging cohort where chronological age, limited to a relatively narrow range of 65–80 years, has partially decoupled from biological aging. These participants represent the segment of the population that has managed to preserve function through lifestyle factors, effectively flattening the expected slope of decline within this specific age window. Furthermore, although the expected mediation pathways were not observed, the direct associations between lower limb strength and TUG performance provide a compelling message for sports medicine practitioners. It suggests that once muscle strength creates a functional deficit, the impact on functional mobility may be biomechanically absolute. Regardless of sex, a weak muscle struggles equally to propel the body mass against gravity. This universality underscores the importance of strength training as a cornerstone of public health guidelines for older adults, irrespective of sex [12].

### Limitations and Agenda for Future Research

The interpretation of these findings warrants the acknowledgement of specific constraints. First, the cross-sectional design precludes the establishment of causal temporality between aging, changes in body composition, and functional decline. Mediation analysis inherently assumes a temporal sequence (i.e., chronological age driving changes in the mediators, which subsequently drive functional mobility decline). Because our data were collected at a single time point, the directional pathways tested in our model must be interpreted as associations rather than proven causal mechanisms. While our moderated mediation model is theoretically grounded in the biomechanics of sarcopenia, longitudinal designs are required to confirm the true mediating sequence of these variables and to determine whether the sex-specific trajectories observed here hold true over time or represent a cohort effect. Second, a limitation lies in the healthy survivor effect potentially introduced by our rigorous exclusion criteria and sampling strategy. By recruiting active community-dwelling older adults and excluding those with neurological conditions or the inability to ambulate independently, we likely selected a cohort of successful aging. This selection bias may explain why age was not a significant predictor of lower limb strength or BMI in our model, as the frailest individuals, those who would drive the expected age-related decline, were effectively screened out. Consequently, our results likely underestimate the magnitude of the associations that would be observed in a frailer or institutionalized clinical population. Third, the unequal sex distribution in our sample (273 females versus 135 males) must be acknowledged. While common in community-based gerontology research, and despite our overall model being adequately powered, this numerical imbalance may affect the robustness and sensitivity of the sex-based moderation analysis. Fourth, regarding anthropometry, while BMI is a widely used epidemiological tool, it lacks the sensitivity to distinguish between adipose tissue and lean muscle mass. The mechanical constraint identified in our study could be further elucidated in future investigations using bioelectrical impedance or dual-energy X-ray absorptiometry to explicitly categorize sarcopenic obesity phenotypes. Furthermore, the use of a stopwatch-based TUG provides a global temporal metric but fails to isolate specific biomechanical deficits. As a result, our data do not allow us to clarify which individual movement components contribute most to the observed sex differences. We could not determine whether the slower performance associated with age in older females was driven by the sit-to-stand phase, gait speed, or the turning phase, a critical component often linked to vestibular function. Fifth, we did not control for or quantify participants’ physical activity levels outside of the structured community programs, which may introduce unmeasured confounding variance in functional outcomes. Finally, while basic cognitive capacity was an inclusion criterion, we did not employ standardized clinical tools to objectively assess cognitive status. Given that cognitive domains, particularly executive function, are known to significantly influence TUG performance, this represents an unmeasured variable that should be accounted for in future models.

The distinct direct effect of age on TUG performance observed in females opens a critical avenue for future investigation. Future studies should prioritize utilizing wearable inertial sensors during the TUG to decompose the movement phases. This would clarify if the female-specific decline is driven by reduced turning angular velocity or linear acceleration. In addition, future studies could incorporate assessments of fear of falling, vestibular function, and executive cognitive function to explain the residual variance in functional mobility decline that strength and BMI failed to capture in women. Last, tracking the transition from robust to frail status to determine the precise age threshold where male physiological reserve typically depletes, potentially revealing a delayed onset of the direct age-balance relationship observed in women, may be of utmost importance.

## 5. Conclusions

This study demonstrates that while lower limb strength and BMI are strong, independent determinants of functional mobility for older adults, they do not mediate the age-related decline in agility within this specific active cohort. The aging process may impact functional mobility similarly across sexes, although conditional analyses hint at subtle sex-specific nuances that warrant further investigation. These findings have important implications for the prescription of exercise and health policies advocated by public organizations, particularly for active, community-dwelling populations. While the promotion of strength training and weight management remains imperative for all older adults, the conditional direct effect of age observed in females suggests a potential avenue for sex-specific adjuvant interventions, provided these differences are confirmed in future adequately powered longitudinal studies. For older females, our conditional findings generate the hypothesis that protocols focusing exclusively on strength may be insufficient to fully mitigate the age-related decline in agility. Although our current data cannot directly substantiate specific clinical prescriptions, these results suggest that future research should explore whether multicomponent programs incorporating vestibular training, dual-task paradigms, and confidence-building strategies are effective in addressing the hypothetical non-muscular components of functional mobility loss that appear to be accelerated in the female aging process. However, given the healthy survivor bias inherent in our study design, these practical recommendations must be interpreted cautiously, as the specific trajectories of functional decline and the required interventions may differ substantially in frailer or institutionalized older adults.

## Figures and Tables

**Table 1:** Descriptive statistics and Pearson correlation coefficients for male and female participants.

Variable	Mean	SD	Min	Max	1	2	3	4
Male								
1. Age (years)	72.60	3.94	66.00	80.00	—	0.045	0.054	0.092
2. BMI (kg/m^2^)	27.04	2.98	21.00	38.00		—	−0.361 **	0.486 **
3. Chair Stand (rep)	14.39	2.97	8.00	21.00			—	−0.516 **
4. TUG (sec)	6.18	1.57	4.38	12.82				—
Female								
1. Age (years)	71.60	3.91	65.00	80.00	—	0.001	0.053	0.113
2. BMI (kg/m^2^)	27.24	3.38	21.00	38.00		—	−0.229 **	0.471 **
3. Chair Stand (rep)	14.52	3.53	8.00	26.00			—	−0.450 **
4. TUG (sec)	6.31	1.48	4.37	13.73				—

Note: SD = Standard Deviation; BMI = Body Mass Index; TUG = Timed Up and Go; rep = repetitions; sec = seconds. ** *p* < 0.01.

**Table 2:** Moderated mediation results predicting functional mobility.

Predictors	*b*	SE	95% CI	*p*-Value	R^2^	F	*p*
Model 1: Predicting Chair Stand (M1)					0.0032	0.43 (3, 404)	0.731
Constant	0.0015	0.1675	[−0.3277, 0.3307]	0.9929			
Age	0.0455	0.0425	[−0.0381, 0.1291]	0.2853			
Sex	0.1766	0.3572	[−0.5256, 0.8787]	0.6213			
Age × Sex	0.0068	0.0902	[−0.1705, 0.1840]	0.9402			
Model 2: Predicting Body Mass Index (M2)							
Constant	−0.0075	0.1626	[−0.3271, 0.3122]	0.9633	0.0013	0.18 (3, 404)	0.909
Age	0.0117	0.0413	[−0.0695, 0.0928]	0.7775			
Sex	0.2146	0.3468	[−0.4671, 0.8964]	0.5363			
Age × Sex	−0.0339	0.0875	[−0.2060, 0.1382]	0.6985			
Model 3: Predicting Timed Up and Go (Y)					0.3699	33.55 (7, 400)	<0.001
Constant	6.2682	0.0604	[6.1495, 6.3868]	<0.001			
Age	0.0466	0.0153	[0.0164, 0.0767]	0.0026			
Sex	0.1712	0.1289	[−0.0821, 0.4245]	0.1847			
Age × Sex	0.0109	0.0326	[−0.0531, 0.0749]	0.7369			
Chair Stand (M1)	−0.1732	0.0190	[−0.2106, −0.1358]	<0.001			
Chair Stand × Sex	0.0569	0.0435	[−0.0286, 0.1424]	0.1914			
Body Mass (M2)	0.1722	0.0194	[0.1340, 0.2104]	<0.001			
Body Mass Index × Sex	−0.0079	0.0438	[−0.0939, 0.0782]	0.8574			

Note: *b* = unstandardized regression coefficient; SE = standard error; 95% CI = 95% confidence interval; M1 = Timed Up and Go; M2 = Body Mass Index. R^2^ represents the proportion of variance explained by each model.

## Data Availability

The data presented in this study are available on request from the corresponding author, provided there is a clear and justified research objective. The data are not publicly available due to privacy and ethical restrictions regarding participant anonymity.
